# Production of Referring Expressions for an Unknown Audience: A Computational Model of Communal Common Ground

**DOI:** 10.3389/fpsyg.2016.01275

**Published:** 2016-08-31

**Authors:** Roman Kutlak, Kees van Deemter, Chris Mellish

**Affiliations:** Natural Language Generation Group, Computing Science Department, University of AberdeenAberdeen, UK

**Keywords:** generation of referring expressions, computational model, common ground, audience design, corpus

## Abstract

This article presents a computational model of the production of referring expressions under uncertainty over the hearer's knowledge. Although situations where the hearer's knowledge is uncertain have seldom been addressed in the computational literature, they are common in ordinary communication, for example when a writer addresses an unknown audience, or when a speaker addresses a stranger. We propose a computational model composed of three complimentary heuristics based on, respectively, an estimation of the recipient's knowledge, an estimation of the extent to which a property is unexpected, and the question of what is the optimum number of properties in a given situation. The model was tested in an experiment with human readers, in which it was compared against the Incremental Algorithm and human-produced descriptions. The results suggest that the new model outperforms the Incremental Algorithm in terms of the proportion of correctly identified entities and in terms of the perceived quality of the generated descriptions.

## 1. Introduction

A large body of research in psycholinguistics investigates the extent to which speakers tailor their utterances to their addressees, a phenomenon known as *audience design* (Clark and Murphy, 1982; Clark and Wilkes-Gibbs, 1986b; Isaacs and Clark, 1987; Clark and Brennan, 1991). Referring expressions (henceforth, res) are a natural focus for research on audience design, because they aim to identify a referent uniquely for an audience; if the re includes information unknown to the hearer, then the hearer may fail to know what or who the speaker talks about. To borrow an example from Appelt (1985), if I tell you to get off the bus “one stop before I do,” then my reference to the bus stop will tend to misfire, because you do not know where I will get off the bus. The link between knowledge and reference makes res a suitable focus for research on Audience Design. The present article follows this well-trodden path, using computational models, and experiments with human participants. Computational models will be employed because they are the most explicit and detailed models of reference production that are currently on the market (see controlled experiments with human participants will help us ground our computational model in actual human behavior.

Audience design is difficult at the best of times. This article focusses on a class of situations in which the process is complicated further by the fact that the speaker addresses an unknown audience, for example as when a novelist writes a book, or a scientist addresses a conference. In these situations, the speaker/writer does not know exactly who is reading or listening, let alone what the listeners know; moreover, different listeners know different things, hence a re that works well for one listener might work badly for another. For concreteness, we focus on res that serve to identify personalities in the public domain (i.e., famous people); generalizations to other publicly known entities—such as companies, towns, sports clubs, and so on—suggest themselves naturally.

Thus, this article presents a computational model of reference to famous people, under uncertainty about the hearer's knowledge. As will be explained, our model rests on three factors. The first is the likelihood that a given property of the referent is known; we call this the **Knowledge** factor. The second is the degree to which a given property is distinctive or useful; we will call this the **Unexpectedness** factor. The third is the completeness of the re; for reasons that will become clear later, we call this the **Termination** factor. These three factors have never before been combined yet they bear important conceptual similarities to each other. For example, just as it is is important for a speaker to know what her audience knows, it is important to know what information is *useful* to her audience, and what amount of it suffices. In the last analysis, these factors might all be seen as part of what theoreticians have called **Common Ground** (e.g., Clark and Marshall, 1981; Clark, 1996; Beaver, 1997; Vanderschraaf and Sillari, 2009).

In the next section, we review the state of the art in computational models of referring, and the extent to which these models are able to capture the insights that have emerged from psycholinguistic (Section 2). Next, we briefly sketch an elicitation experiment that provides us with a corpus of human-produced res 3, allowing us to make some initial observations^1^. Our computational model is presented in Section 4; it is experimentally tested in Section 5 and the results are reported in Section 6. The paper concludes with a discussion of the wider implications of our findings (Section 7).

## 2. Computational models of referring and audience design

Computational models of reference production are also known as referring expression generation (REG) algorithms. Early REG algorithms were, first and foremost, components of dialogue systems (e.g., Winograd, 1972), where they ensure that entities are described in ways that are intelligible to users. Early REG algorithms were not informed by extensive experimentation with human participants. Over the years, however, there has been a gradual shift. First, computational linguists started to incorporate some psycholinguistic findings, hoping this would help them to create more effective referring expressions^2^. Soon after that, REG algorithms started to be tested systematically, for example in terms of the extent to which their output resembles referring expressions produced by human speakers (Passonneau, 1996; Gupta and Stent, 2005; van Deemter et al., 2012). Essentially, this meant that REG algorithms were starting to be seen as *product* models of human behavior, that is, models that focus on the relation between inputs (i.e., the domain and the intended referent) and outputs (i.e., the semantic content of the referring expression), without making further claims about the production *process* (Sun, 2008). Recent REG algorithms are trying hard to simulate human reference production, by modeling phenomena such as variation in language production (Viethen and Dale, 2010; Frank and Goodman, 2012; van Gompel et al., 2012).

In what follows, we first summarize how Audience Design has been understood by theoreticians, and what psychological experiments have taught us about this phenomenon. Next, we discuss to what extent existing REG algorithms address Audience Design. After that, we turn to the challenge outlined in Section 1, namely to model the problem of Audience Design under uncertainty concerning the hearer's knowledge.

### 2.1. Audience design in human reference production

Much of our understanding of reference production is based on the idea of Information Sharing (van Deemter, 2016). To convey the idea using a simple example, suppose our shared information is represented in the Knowledge Base of below table. Suppose, furthermore, I have new information for you about the animal *a*: for example, that it is *in a cage*. To communicate this new information to you, I can exploit our shared knowledge, telling you, “the Kenyan lion is in a cage.”

**Table:** 

**Identifier**	**Species**	**Origin**	**Weight**	**Injuries**
*a*	Lion	Kenya	102 kg	Paws, teeth
*b*	Lion	China	100 kg	Paws
*c*	Tiger	China	310 kg	Back

After my utterance, my *privileged* information has shrunk, but our *shared* information has increased because the fact that *a* is in the cage is now part of it. Crucially, a different re might have been chosen, e.g., “The lion that weighs 102 kg.” The choice of referring expression is what reg algorithms are concerned with.

Most authors on reg have written about Information Sharing as if it hinged on what knowledge the speaker knows the hearer to possess. Although this is an important part of it, psychologists, logicians, and game theorists have argued that, strictly speaking, information *p* is only shared between a set of agents (it is also said to be “common knowledge,” or “in common ground”) if all these agents know *that p and that p is shared*. To borrow an example from Clark and Marshall, suppose I utter the re “the movie showing at the Roxy tonight” (Clark and Marshall, 1981). If you and I believe this is movie *x*, and I believe that you believe it is *x*, but you believe that I believe it is movie *y* then you will misunderstand me, because you think I'm referring to *y*. A proposition *p* is only shared between you and me if I know that *p*, you know that *p*, I know that you know that *p*, you know that I know that *p*, and so on, using epistemic embeddings of arbitrary depth.

Researchers from a number of disciplines have contributed to our understanding of Information Sharing (see Beaver, 1997 for a survey). The philosopher Robert Stalnaker, for example, thought a felicitous utterance should normally fulfill two conditions: it should be consistent with shared information and it should add new information to it (Stalnaker, 1978). This view has sometimes been challenged (e.g., Lewis, 1979 on the notion of *accommodation*), but it matches the relatively simple situations on which reg research has focussed.

To perform information sharing effectively requires the reader to design her referring expressions in a way that allows the hearer to understand what they refer to, a special case of a phenomenon known as Audience Design. Speakers are not always good at Audience Design. The ability in principle of most adult human speakers to reason about “other minds” is well attested, yet speakers and hearers frequently fail to realize exactly what information is shared between them: the extent to which we are able to assess what information is shared is the subject of the so-called *egocentricity* debate. On one side of the debate are psycholinguists who emphasize shared information and its role in communication (e.g., Clark and Wilkes-Gibbs, 1986a; Brennan and Clark, 1996). On the other side are researchers whose experiments have sowed doubt about people's ability to take their knowledge about other minds into account when they speak or listen (Horton and Keysar, 1996; Keysar et al., 2003; Lane et al., 2006), even in situations where it has been made abundantly clear to each interlocutor what the other one knows. Some “doubters” compare our ability to take other minds into account to a fancy espresso machine that you have been given as a present: you own the machine (i.e., you are able to theorize about other minds), yet you may not use it very often (Keysar et al., 2003).

To date, the debate is unresolved, with different researchers attaching different interpretations to experimental results. For example, a study by Wu and Keysar focussed on speakers' choices between names and descriptions (Wu and Keysar, 2007). Participants were shown unfamiliar complex shapes and they were taught equally unfamiliar names for these shapes (e.g., one was called *Abypit*). The authors found that speakers frequently over-use names, tending to produce names where they should have known that the listener had no chance of knowing what they meant. This appeared to confirm the suspicions of the “doubters.” However, in a recent follow-up, Heller and colleagues re-examined Wu and Keysar's experiment, and concluded that speakers in that experiment were not over-using names at all: when they used unfamiliar names, this tended to be in situations where sufficient other information was available to permit hearers to know what the name referred to Heller et al. (2012). Essentially, Heller et al. argue, speakers were *teaching* hearers the meanings of the name.

Other publications in this area have given rise to similar discussions, with critics arguing that experimental participants had been put in unusual situations (Brown-Schmidt, 2009). Instead of exploring these issues further, let us see how the reference task can be formulated as part of a computational model.

### 2.2. Audience design in existing REG algorithms

In accordance with longstanding usage going back to the work of J.S. Mill in the 19th century, we call the set of elements that have a property *P* the *extension* of *P*, abbreviated [[*P*]]. Given is a finite domain involving a set *M* of entities; what entities *M* contains is shared information between the speaker and the hearer. *M* contains an element *rϵM*, the target referent. Given are also one or more other elements, the *distractors*, and a set **P** of atomic properties, whose extension is shared information between the speaker and the hearer. The reg task may be defined as follows:

*The* reg *task* If there exists a subset {*P*_1_, .., *P*_*n*_} of **P** such that [[*P*_1_]] ∩…∩ [[*P*_*n*_]] = {*r*} (so *r* is the only element in *M* of which each of these *n* properties holds true), then reg needs to find such a set. The algorithm needs to make sure that the properties *P*_1_, …, *P*_*n*_ permit the generation of a re that is optimally similar to res produced by human speakers in comparable situations.

Following Dale and Haddock (1991), both the set of properties {*P*_1_, .., *P*_*n*_} and the re that puts the properties into words is called *distinguishing description*. A distinguishing description is thus a set of properties whose conjunction is true of the referent but not of any other entity in the domain; other entities are called *distractors*. We focus here on algorithms that produce “one-shot” descriptions, that is, which disregard any prior utterances.

Existing reg algorithms have used these ideas in different ways. Some early algorithms generate descriptions that are *minimally* distinguishing (i.e., containing the minimum number of properties), but speakers frequently include additional information (e.g., Levelt, 1989; more recently Arts, 2004; Engelhardt et al., 2006; Koolen et al., 2011). Observations of this kind led to the Incremental Algorithm (IA) (Dale and Reiter, 1995), which assumes the existence an ordered list of attributes, known as a *Preference Order*. The notion of a Preference Order formalizes the idea that some attributes are more likely to be used than others, for instance because they have high utility, or high “codability” (Belke and Meyer, 2002). Color, for example, is thought to have high codability, and this explains the fact that referring expressions frequently contain color in situations where the referent could already be identified by the hearer (i.e., the use of color was logically superfluous). The IA produces different output depending on what Preference Order it uses.

The IA generates a description of a referent *r* in the following way: The algorithm takes the first attribute from the Preference Order and selects the most attribute that removes the most distractors. If the property rules out one or more distractors, it is added to the referring expression; otherwise it is not added, and the next attribute in the preference order is examined. Crucially, the algorithm terminates when properties *P*_1_, .., *P*_*n*_ have been selected such that [[*P*_1_]]∩…∩[[*P*_*n*_]] = {*r*}. In other words, the algorithm ends *when the algorithms calculates that the hearer is able to identify the referent*, and this is where, arguably, it performs Audience Design. When the algorithm terminates, the description resulting from it is inspected to see whether another property needs to be added: if the description does not contain a property whose attribute is type, one such property is added to ensure that the description contains a noun.

#### 2.2.1. Example

Suppose a domain contains three chairs *a, b, c*, whose color and size are defined. Suppose *a* and *b* are red, while *c* is brown. Furthermore, *a* is large, whereas *b* and *c* are small. Suppose *a* is the referent, and the Preference Order is [color, size]. Then the IA starts examining the most highly preferred attribute, color, selecting *red*. Although this property rules out the distractor *c*, it does not rule out *b*, so the referring expression is not finished yet, and another property needs to be selected. The next property selected is *large*, ruling out the distractor *b*. Both distractors have been ruled out now, and the type of the object is added, that is *chair*. Later processes decide what words to employ for expressing these three concepts, as in “the large red chair.” Neither is a *minimally* distinguishing description, since “the large chair” would have been unambiguous.

Dale and Reiter hinted at something like Audience Design (without using the term). Their idea was that when the IA asks whether a property rules out any distractors, the answer is given on the basis of *what the speakers believes to be the hearer's knowledge* about each domain object (Dale and Reiter, 1995, Section 2.3). The IA does not offer a mechanism for assessing the hearer's knowledge. In practice, when implemented, the algorithm invariably uses a simple database of facts (as in our Example above). Clearly, it was not the authors' aim to offer an account of Common Ground.

A model that considers the hearer's knowledge to a slightly greater extent is Horacek (2005). Horacek identifies three types of uncertainty: *knowledge, perception capabilities* and *conceptual agreement*. Uncertainty about knowledge occurs when a property may not be known or recognized by the hearer; for example, if the speaker says “*the Basset Hound*,” the hearer may not be able to tell a Basset Hound from other dog breeds. Uncertainty about perception arises, for example, if the hearer does not view a scene from the same position as the speaker, so some properties (e.g., a dog's tail) might be hidden from view. Conceptual agreement uncertainties occur when there is a chance that the speaker conceptualizes a property differently from the hearer; for example, the speaker might describe an object as *turquoise* whereas the hearer would describe it as *blue*.

Horacek (2005) augments the Incremental Algorithm by taking these three types of uncertainty into account. Each property has three probabilities associated with it, one for each of the three types of uncertainty. These three probabilities are combined into one overall probability which helps to determine whether the property in question will become part of the referring expression generated by the algorithm. Although Horacek focussed on very small domains and did not test his algorithm empirically, his work indicates a possible approach to generating definite descriptions under uncertainty. A difficulty is that it is unclear how the necessary probabilities in Horacek's algorithm should be estimated. Our own algorithm (Section 4), by contrast, has computational methods for estimating probabilities at its heart.

### 2.3. Audience design in REG: the challenge ahead

Considerable effort has been invested in experiments that test the ability of REG algorithms to mimic the res produced by human speakers (Gatt and Belz, 2010). Almost invariably, these tests have focussed on communicative situations in which it is straightforward to determine what properties are in the Common Ground of the speaker and the hearer. The typical setup of these experiments has been to elicit res from speakers who are either together in a room with the hearer observing the same visual domain, or they are asked to imagine that they are. Herb Clark described situations of this kind as involving “triple copresence” and observed that these situations make it easy for people to understand what information is in Common Ground.

Although a limited amount of research discusses situations in which the hearer has to make an effort to find out what information is in Common Ground (Garoufi and Koller, 2014a; Paraboni and van Deemter, 2014a), we are unaware of computational models of situations—such as those discussed by Keysar and colleagues—where “egocentric” speakers struggle to realize what is in Common Ground. To mimic speakers' behavior in such situations, a computational model would have to behave as if it has a tenuous grasp of Common Ground, avoiding privileged information in some situations but not in other similar situations; after all, speakers are not unable to understand that the hearer's knowledge differs from their own—they do “get it right” some of the time. To capture this fluctuating behavior, a probabilistic model may have to be designed, which does not always produce the same referring expression in a given situation (Holden and van Orden, 2009; van Deemter, 2016, chapter 6). To do this, however, is not the aim of the present paper.

The problem to which we are about to turn has relevance for the much-debated problem of egocentricity, but instead of examining cleverly designed situations in which speakers *know* very well what the hearer knows but sometimes fail to apply this knowledge, we study the situations discussed in Section 1, where speakers do not have sufficient information to judge what their hearers know. As the domain of our study, we chose the domain of *famous people*. This vast domain forces speaker to guess what properties are likely to be known by hearers. Moreover, the naturalness of the domain allows experimentation with participants without any special training. Finally, this is a domain for which some computational resources exist (such as DBpedia, see Section 4.2) that will prove to be important for the construction of our model.

Our approach owes a debt to Clark and Marshall's insight, that people manage to communicate even in the absence of triple co-presence. These authors suggested that two mechanisms can help us to estimate Common Ground. The first mechanism operates when information is publicly announced, and is called Linguistic Common Ground. For example, when information is broadcast on a train, then this information is accessible to all passengers, so it might be reasonable to assume that it is on Common Ground (barring background noise and lack of attention). The second mechanism operates when people from the same community are exposed to broadly the same sources of information. Residents of Paris, for example, expect other residents to know where the Eiffel Tower is, and they expect other residents to know that they know this, and so on. In connection with situations of this kind, Clark and Marshall coined the term *communal* Common Ground (Clark and Marshall, 1981; Clark, 1996): “common ground based on community membership.”

Linguistic and communal Common Ground can only *estimate* Common Ground, because they do not offer an absolute guarantee that each member of the community knows that each member of the community possesses the information involved. Estimation is, accordingly, an important feature of the computational models that will be discussed later in this paper. These models will focus on *communal* Common Ground. The reader may recall that we are focussing on referring expressions that refer in one shot (i.e., disregarding linguistic context). Algorithms that produce REs in linguistic context might be seen as modeling *linguistic* Common Ground; of particular interest in this connection are models in which an algorithm similar to the ones discussed in Section 2.2 are applied to situations in which the shared knowledge base is essentially a piece of text (Siddharthan and Copestake, 2004).

## 3. Initial exploration: eliciting a corpus of reS

To gain an initial insight in the descriptions produced by human speakers, we elicited a corpus of descriptions of famous people (Kutlak et al., 2011), using Amazon Mechanical Turk (MTurk), a platform where tasks can be posted that are completed by volunteers for a small financial reward. Participants were told about a game where a speaker produces descriptions of a famous person and a hearer has to guess the name of the person described. Participants were told that the hearer has one attempt to guess the name of the person described and the descriptions produced should help the hearer to identify the described person. Participants were then presented with names of famous people and primed to produce definite descriptions by completing the sentence, “This person was the…”. Table 1 shows some of the 215 descriptions produced by 29 participants. Participants were self-identified native English speakers whose registered address was in the US or the UK (see Kutlak et al., 2011 for more details).

**Table 1:** **A sample of referent names and corresponding descriptions in the corpus**.

**Name**	**Description**
Albert Einstein	This person was the author of the theory of relativity
	This person was the physicist who developed the Theory of Relativity that revolutionized how we understand space, time, and gravity
	This person was the German-American mathematician
Thomas Edison	This person invented the light bulb
	This person was most famous for his inventions of the light blub and the phonograph
	This person was the inventor of the light bulb, phonograph, and movie projector
Elvis Presley	This person was the King of Rock “n” Roll
	This person was the King of Rock and Roll, born in Tupelo, Mississippi, who had Graceland built
	This person was one of the most popular singers ever, with hits including Blue Suede Shoes and Jailhouse Rock

Informal analysis of the corpus suggested to us that many descriptions of the same person share a common core of properties. For example, all descriptions of *Edison* said he invented the light bulb; half of the descriptions of *Hillary Clinton* mentioned that she was a former First Lady. Far from being idiosyncratic, the bulk of the properties employed seemed to be ones that are widely known. This observation set us on a path to designing a **knowledge heuristic**, which estimates what information people are likely to know (Section 4.1).

Secondly, many of the properties mentioned in a description were quite unusual with respect to the general population. Examples of such properties are *the inventor of the telephone* and *received a Nobel Prize*. This is to be expected, because participants were asked to produce descriptions that allow hearers to guess the name of the person described. However, it also suggests that if one wants to simulate human behavior, one might take into consideration how unusual a property is. In other words, although properties should be widely known, they should also be unusual or unexpected. This suggests a second heuristic, for which we use the term **unexpectedness**. In other words, we hypothesized that descriptions should avoid properties that are unexpected yet little known (e.g., *the Warden of the British Royal Mint in 1696*^3^), but also properties that are widely known but too common to be useful (e.g., *the person who had arms and legs…*).

Finally, many descriptions in the corpus were multiply over-specified in the sense that they contained multiple properties that could be omitted without stopping the description from being distinguishing. For example, the description “*This person was the author of The Old Man and the Sea, The Sun Also Rises, For Whom the Bell Tolls, and other famous novels”* contains three properties, each of which identifies *Hemingway* uniquely. Perhaps because participants did not know what the hearers knew, they included extra properties to increase the chance that the hearer identify the referent. To produce a minimally distinguishing description would be to gamble, but it is not obvious *how much* over-specification is required. We use the term **termination** to refer to the factor determining how long the reg algorithm should continue adding properties to the description.

## 4. The computational model

Reflecting the insights of the previous section, our computational model of reference production is composed of three heuristics, each of which corresponds to one of the factors discussed above. This heuristic-based approach makes the model transparent, because one can see what each heuristic contributes (though of course we have no evidence that they have separate reality in the human mind). Having individual heuristics also makes the model more extensible, because new heuristics can easily be added. For example, the model could be extended by a heuristic that takes into account the context in which the description appears.

A general remark about the way in which our three heuristics were developed is in order. There are many ways in which the loosely explained ideas of the previous section may be turned into precisely defined metrics that can be applied to actual data. The danger of testing hypotheses involving a large number of metrics is that the risk of type I errors (false positives) is considerable. Our solution to this problem was to use a two-stage approach. During the first stage, we implemented a number of metrics that appeared to be plausible on the basis of earlier work, and we did a pilot test on all of them. During the second stage, those metric that performed best during the pilot were tested again, using a new set of stimuli. Any metrics that achieved a good performance by a chance during the first stage are likely to fail during the second. This approach allowed us to test a large number of options while keeping the risk of type I errors low.

### 4.1. Knowledge heuristic

In order to generate useful descriptions of people, the computational model should select properties that are known by the hearers, and this involves estimating hearers' knowledge. We take as our starting point the idea that speakers use community membership to estimate what hearers know (Clark and Marshall, 1981). Experimental evidence shows that speakers are often able to distinguish between knowledge that is available to members of specific communities or to outsiders. For example, Krauss and Fussell (1991) cite an experiment by Kingsbury (1968), who asked random pedestrians in Boston for directions to a local department store. Kingsbury asked one third of his subjects (a) “Can you tell me how to get to Jordan-Marsh?” using a local dialect, one third (b) “I'm from out of town. Can you tell me how to get to Jordan-Marsh?” using the same dialect, and one third (c) “Can you tell me how to get to Jordan-Marsh?” using his native rural Missouri—a dialect not often heard in Boston. Respondents in groups (b) and (c) provided longer and more detailed responses than in group (a). Related conclusions can be drawn from Bromme et al. (2001) and Nickerson et al. (1987).

Our hypothesis is that the knowledge of a community can be estimated by examining documents produced by the community: the more frequently a fact is mentioned, the more likely are the members of the community to know this fact. This may be motivated by two considerations: firstly, an author who reports a fact will tend to know this fact; secondly, if a fact is recorded frequently, then it may be read often, making it more likely to be remembered (Atkinson and Shiffrin, 1968).

Given our hypothesis, two additional things are required: (a) a corpus of documents that represents the target community, and (b) a metric that allows us to calculate how likely it is that addressees know a given fact. As our corpus, we used the World Wide Web, and to gain information from it we used the search engine Google. The World Wide Web has been successfully used as a corpus before (e.g., Turney, 2001; Keller and Lapata, 2003). The advantage of using a search engine such as Google is its ability to take synonyms and morphological variations into account and to ignore irrelevant words that separate the search terms. Since our queries will use English search terms, the documents retrieved by the search engine are also in English, so we hypothesize that they represent the community of those English speakers who regularly access the World Wide Web.

To implement the Knowledge Heuristic, we experimented with a number of computational metrics of co-occurrence based on the counts of documents containing certain facts, or metrics based on probabilities derived from these counts. Below, we list the four metrics that performed best in our pilot (“stage 1”) experiment, all of which are existing metrics for measuring the strength of association between words. Each of the metrics assumes that a *context* for the words has been defined. These contexts are often defined as a limited number of words before or after the target word or a short frame such as a paragraph in which the target word occurs, but this is not suitable for our purpose, because a fact about a person can be mentioned further away from the person's name, especially if the name is pronominalized in consequent paragraphs. Therefore, we used as our context the entire page returned by the search engine.

#### 4.1.1. Frequency

The simplest measure of association between a person and a property (a fact about a person) is the frequency of occurrence of the name and the property together in a corpus. Taking a collection of documents as a corpus, frequency corresponds to the count of articles that contain the name and the property. This association is then the value of *count*(*n, p*) where *n* stands for the name of an entity and *p* is the property in question.

#### 4.1.2. Conditional probability

More sophisticated measures are conditional probabilities as in Equations (1) and (2), where Equation (1) measures the probability of occurrence of the name given that a property occurs, and Equation (2) measures the probability of occurrence of a property given that the name of the person occurs. While the former measure normalizes the results by the frequency of the property, the later measure takes into account how famous each person is.

(1) $$assoc_{prob}(n,p)=P(n|p)=\frac{count(n,p)}{count(p)}$$

(2) $$assoc_{prob}(p,n)=P(p|n)=\frac{count(p,n)}{count(n)}$$

#### 4.1.3. Pointwise mutual information

(PMI) Fano (1961) compares how often two events *x* and *y* occur together. PMI exploits the fact that if two terms appear together often, their joint probability (*P*(*n, p*)) will be higher than if they were independent (*P*(*n*)*P*(*p*)). The value of PMI is positive for terms that co-occur and negative otherwise.

(3) $$assoc_{PMI}(n,p)=log_{2}\frac{P(n,p)}{P(n)P(p)}$$

A problem with PMI is that infrequent words that only appear together achieve a disproportionately high score. This is undesirable, because in order for a property to be in common ground, it also has to be frequently mentioned. To mitigate this type of problem, Hodges et al. (1996) suggest multiplying each PMI score by *count*(*n, p*). To balance out the large differences between the frequencies of frequent items (hundreds of thousands of results) and less frequent items (hundreds of results), we multiply the PMI score by the square root of the count. The final formula used for calculating the association is as in Equation (4).

(4) $$assoc_{PMI}(n,p)=\sqrt{count(n,p)}*log_{2}\frac{P(n,p)}{P(n)P(p)}$$

These four metrics were first tested in a pilot experiment and the best performing metric was then re-tested (in what we call the “main experiment” in this section) using a different set of stimuli.

Given that the setup of the pilot and the main experiment was essentially identical, we describe the the method and the procedure only once. Participants, materials, and results are reported separately for both the pilot and the main experiment.

#### 4.1.4. Materials and method of the pilot experiment

**Figure 1:**
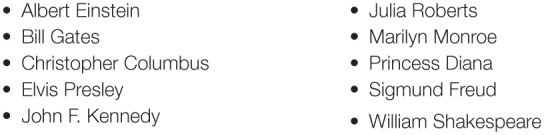
**Names of famous people used in the pilot test of the potential metrics for the Knowledge Heuristic**.

For the pilot experiment, we selected 10 people, each of whom was famous enough that his/her name occurred on the BBC Historical Figures page^4^. The 10 people were selected in such a way that they varied in terms of how well known they were likely to be. The names of the selected people are listed in Figure 1.

For each referent we selected information from Wikipedia and the BBC Historical Figures pages. We used our own judgment (informed by the frequencies of properties from the corpus described in the previous section) to select properties that covered a range of likelihoods of being known. That is, for each referent, we selected a number of properties that were likely to be known by anyone who knows the referent (e.g., the referent's occupation), and properties that were likely to be known only by people who have a more detailed knowledge of the referent.

For each referent, we included 5 filler properties that were not true of the referent. Each trial contained 5 true properties and 5 filler properties for each person, presented in randomized order. This resulted in a total of 100 statements (10 referents, 10 properties each). To keep the task manageable, statements were randomly split into 5 groups of 20 statements.

#### 4.1.5. Participants and procedure of the pilot experiment

The pilot experiment was conducted online using Amazon Mechanical Turk (MTurk) to ensure a large number of participants. A total of 755 MTurk users started the experiment, but only 216 completed the experiment^5^. A further 12 were removed because the number of errors (counted as answering “true” to a false statement or vice versa) was higher than 5 (avg. + SD). The resulting dataset contained 4080 answers produced by 204 participants (78 female, 126 male).

The first screen showed instructions on how to answer and how to navigate the website and also urged the participants to rely on their own knowledge and avoid using external resources to answer the questions. The participants were then asked to fill in some information such as their sex, age group and interests. The participants were then randomly assigned to one of the groups. The participants viewed one statement at a time and were asked to select one of the options (true, don't know, false). Participants could also add a comment to each statement; at the end of the experiment they were given an opportunity to offer additional open comments.

#### 4.1.6. Results of the pilot experiment

The responses from participants were aggregated per statement, resulting in each statement having a percentage of affirmative answers (answers where participants selected true). Only true statements were analyzed. Table 2 shows example statements and their scores for Albert Einstein.

**Table 2:** **List of properties true of Albert Einstein**.

**Property**	**Percentage**	**Frequency**	**Rank**
Albert Einstein was a physicist	80.95	827000	4.00
Albert Einstein invented the theory of relativity	80.43	69600	3.00
Albert Einstein was German	67.39	1060000	5.00
Albert Einstein emigrated to the United States	47.06	20200	2.00
Albert Einstein was a professor at the Karl-Ferdinand University in Prague	30.30	652	1.00

The percentage of affirmative answers show what percentage of participants believed the statement to be true. Rank shows how the corresponding properties ranked according to the Knowledge Heuristic. Spearman correlation between percentage and frequency r_s(48)_ = 0.67;p < 0.001.

Table 3 shows correlations between the metrics and the percentages of affirmative answers calculated from participants' answers. Frequency, *p*|*n* and PMI performed very well. Given the simplicity and the performance of the Frequency metric, we decided to chose this metric for the Knowledge Heuristic, where its performance in conjunction with the other metrics was to be tested.

**Table 3:** **Pilot results: Spearman correlation between the metrics and knowledge of hearers**.

	**Frequency**	***n*|*p***	***p*|*n***	**PMI**
*rs*(48)	0.667	−0.063	0.672	0.628
*p*-value	0.000	0.663	0.000	0.000

#### 4.1.7. Materials of the main experiment

**Figure 2:**
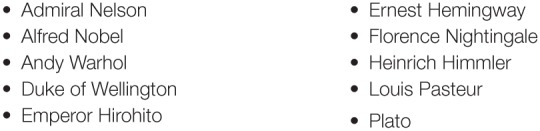
**Names of famous people used in the test of the Knowledge Heuristic**.

Similarly to the pilot experiment, we selected 10 famous figures (see Figure 2), each of whom was famous enough that their names occurred on the BBC Historical Figures page^6^. Each trial contained 7 true properties and 5 filler properties for each person, presented in randomized order. This resulted in a total of 120 statements (10 referents, 12 properties each). The statements were also randomly split into 5 groups of 24 statements (14 true, 10 false in each group).

#### 4.1.8. Participants of the main experiment

The main experiment involving the Knowledge Heuristic was likewise conducted online using Amazon Mechanical Turk (MTurk). The pilot experiment had a large proportion of users from India. Given that the experiment required knowledge of “western” culture, we decided to limit the main experiment to US and UK MTurkers. Furthermore, participants also had to successfully pass a cloze test (Stubbs and Tucker, 1974), guaranteeing that only highly fluent speakers of English would pass.

The main experiment was undertaken by 71 English speakers. 5 were discarded because they did not finish the experiment and a further 5 participants were removed because the number of errors they made was more than 4 (mean + 2 ^*^ SD). The total number of participants was 61; of these, 30 females, 29 males and 2 unspecified.

#### 4.1.9. Results and discussion of the main experiment

Answers were aggregated by statement and the resulting percentages were correlated with scores assigned by the Knowledge Heuristic. Table 4 shows examples of the statements used in the experiment, along with the percentages of answers where participants selected true and the ranks assigned by the Knowledge Heuristic. The search queries were run in June 2014. We found a high correlation between the estimates produced by the heuristic and the percentage of people who knew given facts [*r*_*s*(68)_ = 0.73;*p* < 0.001]. The heuristic performs very well if we compare the results of the heuristic with the correlation of estimated and actual knowledge of human speakers as reported in Fussell and Krauss (1992). In their experiments, Fussell and Krauss report that the average correlation of people's estimate of knowledge of others and their actual knowledge was 0.67 (note that this was a Pearson correlation and our results report a Spearman correlation).

**Table 4:** **List of properties of Ernest Hemingway**.

**Property**	**Condition**	**Percentage**	**Rank**
Ernest Hemingway was a writer	True	100.0	1
Ernest Hemingway was American	True	100.0	2
Ernest Hemingway received the Nobel Prize in Literature	True	63.6	5
Ernest Hemingway is the author of For whom the bell tolls	True	54.5	4
Ernest Hemingway committed a suicide	True	50.0	3
Ernest Hemingway was British	False	27.3	–
Ernest Hemingway was born in Oak Park	True	25.0	6
Ernest Hemingway received the Italian Silver Medal of Bravery	True	20.0	7
Ernest Hemingway is the author of A tale of two cities	False	13.3	–
Ernest Hemingway invented dynamite	False	0.0	–
Ernest Hemingway died in a plane crash	False	0.0	–
Ernest Hemingway was born in Paris	False	0.0	–

Condition shows whether a property was true or false (a filler) and the percentage of affirmative answers shows what percentage of participants believed the statement to be true. Rank shows how the corresponding properties ranked according to the Knowledge Heuristic. Spearman correlation between percentage and rank r_s(68)_ = 0.73;p < 0.001.

These results suggested to us that our Knowledge Heuristic is a viable starting point for a computational model of people's knowledge. Note that, strictly speaking, our heuristic was not tested in terms of its ability to capture Common Ground. After all, for a proposition to be in Common Ground (in the strict sense) between all members of a community, it is not sufficient for the proposition to be known by all members: additionally, all members should know that the proposition is in Common Ground; the recursion in this formulation implies an infinite sequence of epistemic iterations (Section 3). Testing our heuristic's ability to capture classic Common Ground would have been very difficult, which is why we settled for a simpler test. Whether this leads to a heuristic that is useful for our purposes is something we were only able to determine when our complete model was tested (Section 5).

However, the Knowledge Heuristic is not sufficient for producing referring expressions, because it does not take into account whether a piece of information is distinguishing. For example, if the heuristic had to decide between properties such as *X is a scientist* and *X is a physicist*, it would assign a higher score to the former. The next section will discuss a heuristic that will balance this deficit.

### 4.2. Unexpectedness heuristic

The analysis of the corpus showed that some of the properties selected by human speakers are unexpected with respect to the population as a whole (e.g., “*inventor of dynamite,” “received a Nobel prize”*). In order to tell the unexpected properties apart from the more common ones, it would be instructive to look at DBpedia. DBpedia is an ontology derived from Wikipedia, a free encyclopedia created by the community of its users. DBpedia extracts some of the information available as free text in Wikipedia and encodes it in machine-readable form. The data is ontologically structured, for example Physicist is a subclass of Scientist, and Scientist is a subclass of Person; this information would be difficult to infer from free text. DBpedia suits REG algorithms as it describes each entity by means of properties in the form 〈*attribute* : *value*〉. Furthermore, each entity in DBpedia has a type (e.g., 〈*type* : *person*〉 or 〈*type* : *scientist*〉), which is something many REG algorithms require, as we have seen in Section 2.2.

Finding the right unexpectedness heuristic proved to be challenging. We are interested in properties that are unexpected for our audience. For example, being awarded the Nobel prize is unexpected because only a handful of people receive this prize every year; on the other hand, having a mother is expected as everyone has a mother. Some REG algorithms achieve unexpectedness by selecting properties that are highly discriminating, as defined by Dale's Discriminatory Power (DP; Dale, 1992). The DP of a property is defined as (*N* − *n*)∕(*N* − 1) where *N* is the total number of entities and *n* the number of entities with a given property. The result of the function takes values between 0 (the property is true for all entities in the context, hence it is not discriminating at all) and 1 (the property is true for only 1 entity in the context, hence it is highly discriminating).

As we are aiming for interesting properties, rather than distinguishing one, DP seemed to be less suitable. One of the problems with DP is the uniform treatment of properties across entity types. For example, in the case of our people domain, a property such as 〈*Nobelprize* : *literature*〉 has almost exactly the same score for a writer and a physicist, which is undesirable. A second problem with DP relates to the context we used it in. Although DBpedia contains millions of entities, the properties are very sparse, therefore DP scores many properties very highly: the DP tends to place many properties close to 1; as a result, it does not provide a lot of information about these properties.

One field that studies the recognition of unusual patterns is data mining. Geng and Hamilton (2006) performed an extensive survey of statistical measures of interestingness and categorized them into concepts such as *surprisingness, peculiarity, utility, etc.* Surprisingness or unexpectedness was typically defined in terms of contradicting a person's existing knowledge or expectations; formalizations of this idea often make use of conditional probability; for example, winning a Nobel Prize may be unexpected (even) for a physicist because the probability P(Nobel | Physicist) is low.

To test the ability of a metric to measure unexpectedness, we conducted an experiment similar to the one on the *quality* of expressions in Section 5.3. Participants were told: “*Imagine your friend tells you he has read something interesting about a person. He tells you the name of the person, but you've never come across the name, so you ask who this is. Your friend wonders what to tell you about this person. Please rate, for each of the facts below, how interesting you would find this fact. Please rate each fact individually (i.e., in isolation from the other facts in the list).”* We tested over 30 statistical measures from Geng and Hamilton (2006) but our pilot found no reliable correlation between the predictions of a metric and people's judgements.

While we were unable to find a metric that performed well on its own, we were able to use our experience with the 30 existing metrics to construct a new metric that showed good results when combined with the Knowledge Heuristic. The metric described by Equation (5) has a greater range of values than DP, as can be seen from Table 5, assigning higher scores than DP to properties that are less frequent. For instance, in Table 5, 〈type:astronaut〉 is much more unexpected than 〈type:scientist〉 according to this metric than according to DP, which we believe to be as it should be (i.e., intuitively, astronaut is a more interesting property than scientist). Unlike DP, our formula looks at a property *B* of a referent in connection with the *type* of the referent, *A*.

(5) $$Score_{unexpectedness}(A,B)=\frac{P(AB)/P(A)P(B)}{\sqrt{P(A)P(B)P(\neg A)P(\neg B)}}$$

**Table 5:** **Unexpectedness of some properties, by Equation (5) and by DP, calculated across DBpedia**.

**Property**	**Unexpectedness (Equation 5)**	**Discriminatory Power**
〈type:thing〉	0	0.0
〈type:person〉	11	0.6233
〈type:scientist〉	89	0.9962
〈type:astronaut〉	430	0.9998

### 4.3. Termination heuristic

The last heuristic determines how much information should be included in a description. Like most REG models (Section 2.2), our algorithm will add properties one by one. Consequently, the number of properties in the description generated depends on when the algorithm terminates. For this reason, we refer to this as *termination*. As we have seen in the corpus, human-produced descriptions often contain several properties that are uniquely distinguishing on their own. Using the traditional approach of terminating when all distractors are ruled out would never produce such descriptions.

As with other heuristics, we tested several methods for terminating the algorithm. Assuming that documents produced by a community can tell us something about the knowledge of the community, we focused on document-retrieval based methods. The intuition was that retrieving documents that contain properties listed in a description can tell us something about the distractors that the audience is likely to be aware of. For example, if our description contained the properties “*singer”* and “*rock 'n' roll*,” a large number of documents would match this description. If the description also contained the property “*singer of Jailhouse Rock*,” the set of matching documents would be much smaller. Three methods were tested. Each time a property was added to a description, the Google search engine was queried using the new description; the search engine returned the number of results plus snippets of text from each document retrieved.

The first method is based on the frequency with which the name of the referent occurs in the documents retrieved when a given description is used as a query. The algorithm constructs a description by adding properties to it. At each stage, the method focusses on the description under construction and examines the snippets returned by the search engine (given that the description is used as a query) and counts what percentage of the snippets contain the name of the intended referent. If this percentage crosses a threshold, the algorithm terminates; otherwise, a further property is added to the description. The process repeats until the threshold is crossed or the description contains the maximum number of properties. Based on our analysis of the corpus in Section 3, the maximum number we allowed was 7 (average + 2 SD).

The second method uses only the counts of the results (i.e., documents returned) that contain the name of the target referent; let's call this number *N*. As in the first method, every time the algorithm adds a new property to the description, the search engine is queried using that description. The number of results will decrease, since fewer and fewer documents match the query. As soon as the number of results falls below a pre-determined fraction of *N* (or when it reaches the maximum number of properties), the algorithm terminates.

**Figure 3:**
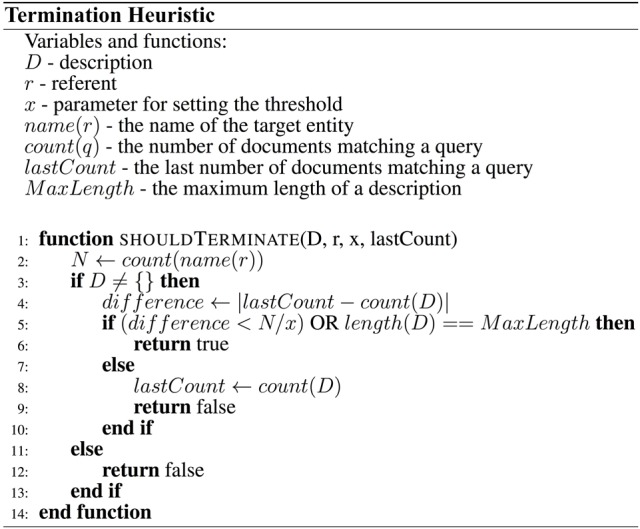
**Pseudocode describing the termination heuristic**. The heuristic returns true (and terminates the algorithm) when adding a property to a description does not result in a large decrease in the number of matching documents.

The third method used the *differences* between the numbers of retrieved documents as a description is being created (i.e., the slope of the graph). Initially, every addition of a property results in a large reduction in the number of results, but as the number of properties in a description increases, the reduction becomes smaller. The heuristic uses this observation to decide whether a description contains enough properties. As soon as the addition of a property does not result in a large difference in the number of matching documents, the heuristic terminates the algorithm. The meaning of “large” is determined by a predetermined threshold. The full heuristic is described by Figure 3.

All thresholds were based on the counts of documents containing the name of the referent and an empirically determined coefficient. The reason for using the number of documents containing the referent is that the amount of content needed to describe a person is related to how well known the person is. It seems plausible that very famous people require fewer properties to identify them than less known people will require. The coefficients were derived from a subset of the corpus described in Section 3 annotated with semantic properties.

The three methods were tested using the corpus of Section 3. Each method took as an input the name of the referent and a list of properties that human participants had written to describe the referent, ordered from most to least frequent. The length of the description produced by each method was compared against the average length of all descriptions of a given referent. The score of the description created by the heuristic was calculated using (Equation 6), a standard *z*-score, where μ_*i*_ is the average length of the descriptions of *i*, and σ_*i*_ is their standard deviation:

(6) $$score(description_{i})=\frac{\left|\mu_{i}-length(description_{i})\right|}{\sigma_{i}}$$

The third method produced the best results, with an average score of 0.98. The average number of properties per description produced by people was 3.349 with *SD* = 1.754 and the average number of properties selected by the termination heuristic was 3.41 with *SD* = 1.34. This heuristic was selected for the final computational model. The heuristic is described using pseudocode in Figure 3.

### 4.4. Combining the three heuristics

**Figure 4:**
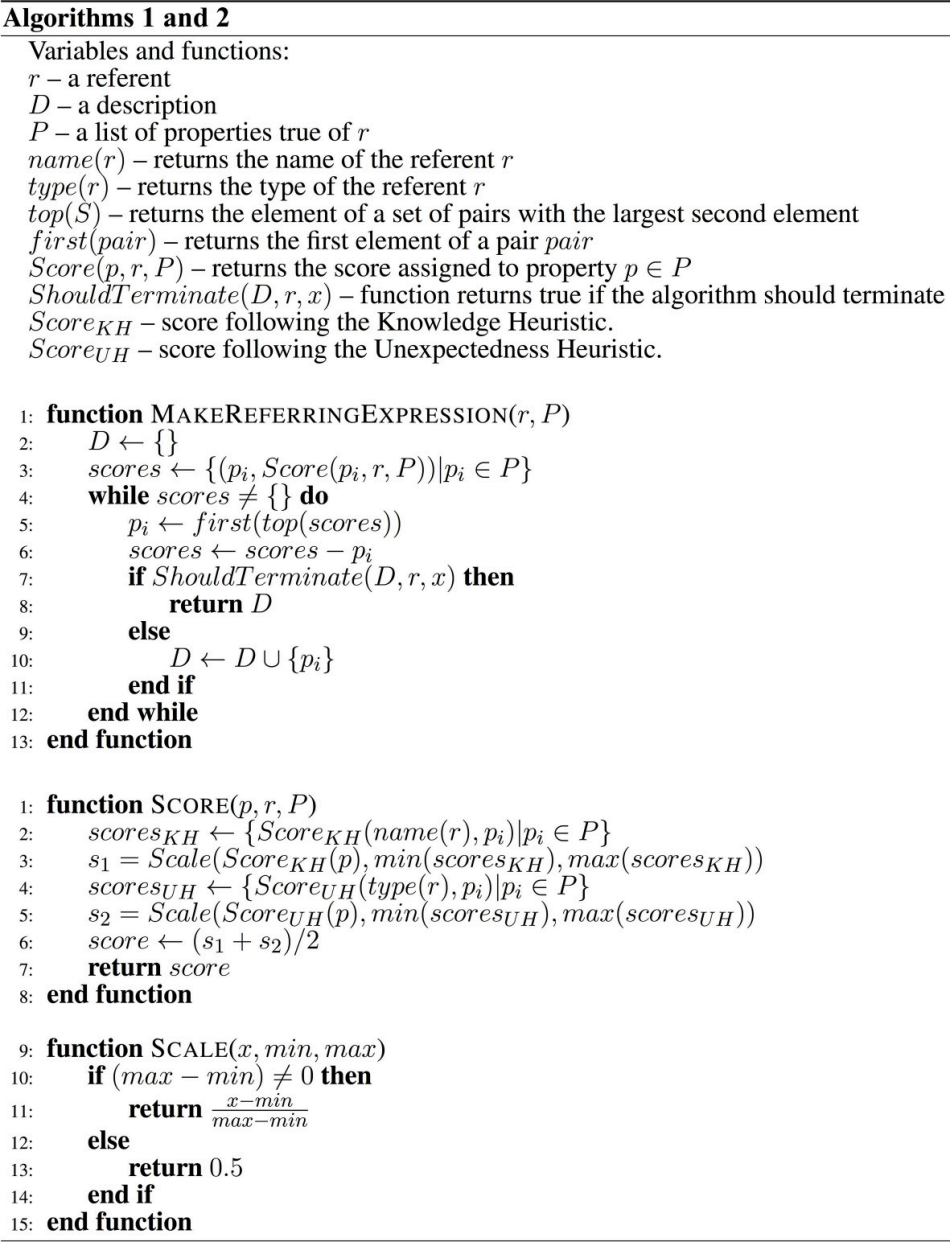
**Pseudocode for algorithms Alg1 and Alg2**.

The above heuristics were combined as in Figure 4. The algorithms start by calling the function MakeReferringExpression and passing as parameters the referent and a list of properties true of the referent. An initially empty list *D* is created, which will later contain the properties used in a description. A score is assigned to each property, based on the combination of the Knowledge Heuristic and the Unexpectedness Heuristic. A loop is then entered where the property with the highest score is taken and removed from the list. Next, it is checked whether the algorithm should terminate. If the Termination Heuristic returns true, the algorithm returns the list *D* as the final description. If the Termination Heuristic returns false, the algorithm adds the current property into the list *D* and loops back to selecting the next property with the highest score. The loop repeats until the Termination Heuristic stops the algorithm.

The score for each property is calculated as follows. Each property is tested by the Knowledge Heuristic and the Unexpectedness Heuristic. The scores assigned by each heuristic are scaled to range between [0–1] using the function *Scale*. The reason for scaling the values is that each heuristic is using a different scale. (Taking an average would not make sense if, for example, the Knowledge Heuristic produced values between 0 and 10^12^ and the Unexpectedness Heuristic would produce scores between 0 and 1). The function for calculating the score of a property is described in Figure 4.

The algorithm is similar in some ways to the Greedy Heuristic (Dale, 1989) because it always chooses the “best” property (based on the three heuristics). Unlike the Greedy Heuristic, our algorithm does not re-calculate the scores of the properties after each iteration. In this regard, the algorithm is more similar to the IA because once the properties are ordered by the heuristics, the order does not change. Unlike the IA, however, the preference is computed for each referent individually.

We also tested a baseline heuristic for terminating the algorithm based on the average number of properties in the human produced descriptions, which is 3. This baseline is not sensitive to the content of the description, risking descriptions that are too general to allow identification. Our experiment in Section 5 therefore tests two different versions of the algorithm: both used the Knowledge Heuristic and Unexpectedness Heuristic to rank the properties but Alg1 used always 3 properties per description whereas Alg2 used the document-retrieval based termination heuristic.

## 5. Evaluating the model

Evaluation of the model focused on three aspects of the descriptions produced. We decided that the main aspect to focus on was the number of successfully identified referents, because identification is the main purpose of referring. The second aspect was *naturalness*, defined by the statement: “*How natural does the description read to you? (For example, could one of your friends produce such a description?)”*. This should tell us something about the human-likeness of the descriptions produced. After all, a description may be effective yet unlike anything that a human speaker is likely to produce. The third aspect was *quality*, defined using the statement: “*Suppose you did not know this person, how good would you find the description? (Does it give a good idea of what sort of person it is or was?)”*. We felt it important to assess this because an addressee may not know the referent, in which case the number of successfully identified referents (our first metric) misses the point.

### 5.1. Algorithms/models considered during evaluation

Computational algorithms were tested along with two types of human-produced descriptions. The first were short descriptions available in DBpedia. Most entities in DBpedia contain the property description, which is comparable to the first line in a Wikipedia article describing an entity. Where the description was not available in DBpedia, we used the first line in Wikipedia (not counting the dates of birth and death).

The second type of human-produced descriptions were created specifically by a native English speaker. A postgraduate student with experience in natural language processing was given a set of 100 names and asked to create English descriptions matching the scenario presented to the participants. That is, the student was asked to produce descriptions of the names so that other US/UK people can guess who the described person is. The student was not aware of the aims of our research but had access to external resources (e.g., the World Wide Web), to ensure that he was able to create descriptions for all entities and not only the ones known to him. We will talk about the “algorithm” DBP when referring to DBpedia descriptions and about the “algorithm” Human when referring to the descriptions produced by the student.

The IA is often used as a reference point against which other algorithms are compared. However, the performance of the IA depends crucially on the chosen preference order. To find a good preference order, we used the semantically annotated part of the corpus described in Section 3. The annotation is similar to the annotation of the TUNA corpus (van Deemter et al., 2012). The preference order was found by taking the annotated properties and ordering them by their frequency from the most to the least frequent attribute. This method has been used by a number of researchers (e.g., Koolen et al., 2012; van Deemter et al., 2012). The first 10 attributes of the preference order were *type, occupation, nationality, country, starring, author, known for, genre, gender, death cause*.

Given the above, we set out to compare 5 classes of descriptions: the ones generated by the algorithms Alg1 and Alg2the one generated by IA (with the preference order as stipulated), and finally the two human-produced descriptions DBP and Human.

“Descriptions” produced by a computer algorithm are nothing more than a list of properties. To allow participants to judge descriptions in a natural way, we felt that these rather formal descriptions had to be converted into real English text. For example, the property 〈writerOf:The Pit and the Pendulum〉 can be written as “the writer of The Pit and the Pendulum.” We created a program that converted properties from DBpedia into English using predefined mappings from properties to strings. All descriptions were post-edited by a native English speaker with experience in linguistics to remove any redundancies in the descriptions and to improve the fluency of the generated descriptions. The English speaker was not involved in the research and had no awareness of its aims.

Our null hypothesis is that “*there are no differences between algorithms in terms of the numbers of correctly identified referents*,” and similarly, “*there are no differences between algorithms in terms of their naturalness and quality*.” We expected the descriptions produced by Human to perform best, as their descriptions are likely to contain enough information to unambiguously identify the referent. The descriptions extracted from DBpedia (algorithm DBP) are likely to perform poorly in terms of identification, as they are often quite general (e.g., “*a famous English writer”*).

### 5.2. Materials for evaluating the model

The names of targets were selected from two websites with lists of names of famous people^7^. We selected 100 names that were not used in our pilot experiments. The evaluation therefore contained 100 names and 500 descriptions in total, given that each referent was described using 5 sources: Alg1, Alg2, IA, DBPand Human.

We used a repeated-measures Latin square design in which each participant viewed a number of descriptions generated by each algorithm (within-subject design). To avoid presenting participants with too many descriptions, the 100 names were randomly assigned to 4 groups of 25. Each group was arranged into a Latin square so that each participant judged 5 descriptions generated by each of the 5 sources (25 descriptions per participant). Furthermore, each description was viewed three times, each time by a different participant.

The order in which descriptions were viewed might bias the results (e.g., seeing a description of Albert Einstein might make it easier to guess Niels Bohr), therefore the order of the descriptions was randomized for every participant. Each description was viewed by three participants.

### 5.3. Participants and procedure for evaluating the model

The evaluation was carried out in accordance with the recommendations of the University of Aberdeen Handbook For Research Governance and approved by the College of Physical Sciences Ethics Review Board. Participants were informed that their participation was completely voluntary and that they could withdraw from the survey at any time for any reason. Participants were informed that the information was used solely for research purposes. No personal information would be shared with any third party. Participants who agreed with the conditions could proceed with the experiment.

The evaluation involved 60 participants (37 male, 22 female and 1 unspecified). In terms of highest achieved education, 26 of the participants had high school, 26 participants had an undergraduate degree and 8 participants had a postgraduate degree. Participants took on average 28 min to complete the task.

Once again, participants were recruited using MTurk. The experiment was advertised only to US MTurk workers who had at least 85% success rate (at least 85% of tasks that a worker submitted in the past were deemed acceptable by the requester). The reason for advertising only to US workers was to maximize the overlap between the knowledge of the participants and the knowledge captured by DBpedia. The famous people employed as target referents are famous in western culture, particularly in the US. The selection criteria ensured that participants were from essentially the same population as the participants who created the corpus of Section 3.

**Figure 5:**
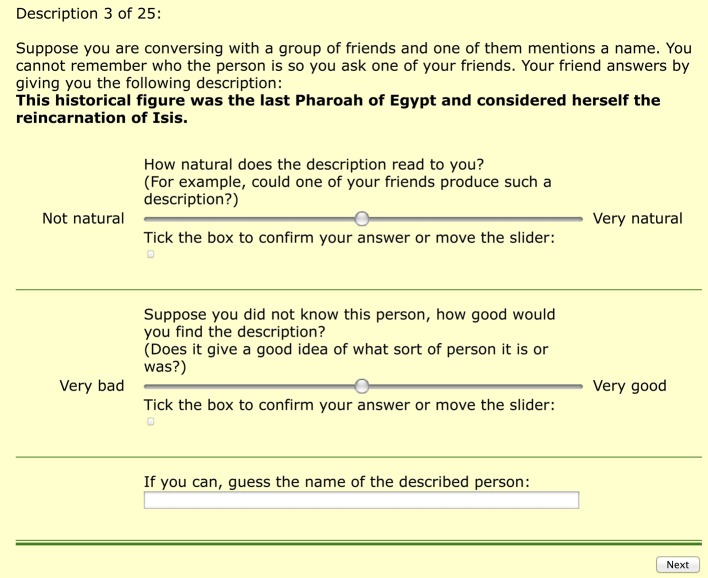
**Presentation of descriptions in the evaluation**. Participants provided judgment of each description by moving the sliders. The box at the bottom of the page was used for providing the name.

Participants were directed to a website that provided instructions. Participants were asked to provide some demographic information (age group, interests, etc.). After submitting this information, participants were shown detailed instructions on how to fill in their answers. After clicking on a button the first description appeared on the screen (Figure 5).

Participants had to judge the description and fill in the name of the referent if they could. The two judgment questions were: “*How natural does the description read to you? (For example, could one of your friends produce such a description?)*” and “*Suppose you did not know this person, how good would you find the description? (Does it give a good idea of what sort of person it is or was?)*”. These two judgments are referred to as *naturalness* and *quality* respectively. Participants provided ratings by moving sliders (similarly to Gatt et al., 2009). The sliders corresponding to each statement were set to the middle position and participants gave their judgment by moving each slider along the horizontal axis. The numerical values corresponding to the sliders were 1–100, but participants were not shown the number. If a participant wished to leave the slider in the middle (value 50), they had to tick the corresponding check box below the slider. This was done to prevent participants from accidentally leaving the slider in its original position without intending to offer a judgment.

**Figure 6:**
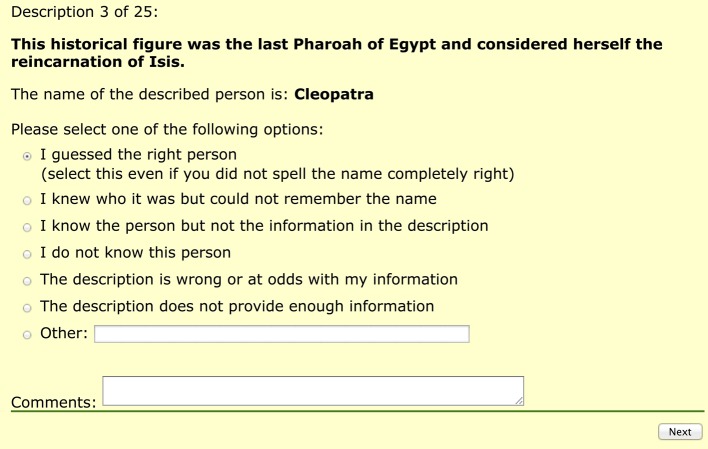
**Options shown to participants after guessing the name of the described person**.

Clicking the button “Next” sent participants to a page with the description and the name of the described person. Participants then had to choose their response from one of the options in Figure 6. The options were mutually exclusive and were used to gain more insight into the features of the descriptions that were produced:

Option 1 indicates successful identification. It was also used to filter out participants who did not take the task seriously. Any participant who provided a wrong name and selected this option was removed from the result set (as they were shown the correct name). The names provided by participants were checked against the actual referent names.

Option 2 was included for participants who experienced the “tip-of-the-tongue” event (ToT, Brown and McNeill, 1966). This option accounted only for about 3% of answers (Kutlak et al., 2013).

Option 3 accounts for situation where the algorithm selects information that is not known by listeners. If an algorithm generates descriptions that frequently lead to this situation, the algorithm is probably selecting properties that are too difficult for people to recall (e.g., dates or numbers).

Option 4 was added to avoid lowering the score of good descriptions where participants do not know the target.

Option 5 covers situations in which some of the properties in the description are not true of the target (i.e., a participant believes to have knowledge that contradicts the information in the description).

Option 6 accounts for situations where an algorithm selects too few properties or where the selected properties are too general. For example, describing a target as *this person is an actor* is unlikely to allow identification of the target.

The participants had the chance to provide any other reason for not being able to identify the target (option 7) as well as providing comments.

## 6. Results of evaluating the model

Table 6 contains frequencies of selected answers for each algorithm. Two answers were not saved due to a technical error. The χ^2^ test compares the observed frequencies with expected frequencies based on the totals in each row and column. In order to focus on the differences in numbers of correctly identified referents, participants' answers were collated into two categories: *correct* and *incorrect*. Responses where participants did not know the referent were removed from the analysis. Where participants selected the Tip of the Tongue (ToT) option, they failed to provide the name of the referent, yet they did have the right person in mind; because this makes it difficult to say whether these answers were correct, we excluded them from our analysis. Table 7 shows the collapsed counts where Incorrect Identification is the sum of the figures in the categories Unknown Properties, Underspecified, At odds with my information, and Other.

**Table 6:** **Counts of selected answers for individual algorithms in the final evaluation**.

	**DBP**	**IA**	**Alg1**	**Alg2**	**Human**	**Total**
Correct identification	68	58	89	100	180	495
Tip-of-the-Tongue (ToT)	21	17	24	24	28	114
Unknown target	44	64	61	61	51	281
Unknown properties	55	136	106	94	33	424
Underspecified	104	21	7	10	2	144
At odds with my information	2	3	8	6	3	22
Other	6	1	5	4	2	18
Total	300	300	300	299	299	1498

DBP are descriptions from DBpedia, IA is the Incremental Algorithm, Alg1 is the new algorithm that always selects three properties, Alg2 is the new algorithm that uses document retrieval as a termination heuristic and Human are descriptions produced by a native English speaker.

**Table 7:** **Counts of correctly and incorrectly identified referents**.

	**DBP**	**IA**	**Alg1**	**Alg2**	**Human**	**Total**
Correct identification	68	58	89	100	180	495
Incorrect identification	167	161	126	114	40	608
Total	235	219	215	214	220	1103
Proportion correct	0.29	0.26	0.41	0.47	0.82	0.45
Proportion incorrect	0.71	0.74	0.59	0.53	0.18	0.55
Correct/incorrect	0.41	0.36	0.71	0.88	4.50	0.81

The table also shows the proportions as well as the ration of correctly and incorrectly identified referents.

As we can see from the table, the descriptions generated by an English speaker outperform every other algorithm. The effect of algorithms was tested using the χ^2^ statistics with the numbers from Table 7. The null hypothesis was rejected as the test showed significant differences: $\chi_{\left(4\right)}^{2}=176.8,p<0.001$. *Post-hoc* pairwise comparison shows statistically significant differences between algorithms IA and Alg2 [$\chi_{\left(1\right)}^{2}=18.3,p<0.001$], IA and Alg1 [$\chi_{\left(1\right)}^{2}=10.1,p<0.005$] and Alg2 and Human [$\chi_{\left(1\right)}^{2}=56.8,p<0.001$].

Even though no description contained the full name of the corresponding referent, some descriptions still contained a “clue” in the form of a property containing a part of the referent's name. For example, *John Lennon* was described as “*This person wrote I Know I Know and was the topic of the musical Lennon.”* In most cases, clues were the names of relatives (e.g., “the spouse of Victoria Beckham,” “relative of Earl Woods”) or names of related entities (“a member of The Jackson 5,” “the creator of The Cosby Show”).

To show the differences between the algorithms more clearly, we removed descriptions of all referents that contained such clues. This was the case for 29 out of the 100 entities and a total of 346 name guesses. Table 8 contains the counts of correctly and incorrectly identified referents on the resulting subset of name guesses. The numbers of correctly identified referents differed significantly between the algorithms tested $\chi_{\left(4\right)}^{2}=119,p<0.001$. *Post-hoc* pairwise comparison resulted in the homogeneous subsets in Table 9.

**Table 8:** **Counts of correctly and incorrectly identified referents when descriptions that contained a clue as to the identity of the referent were removed**.

	**DBP**	**IA**	**Alg1**	**Alg2**	**Human**	**Total**
Correct identification	44	41	49	63	123	320
Incorrect identification	112	110	97	86	32	437
Total	156	151	146	149	155	757
Proportion correct	0.28	0.27	0.34	0.42	0.79	0.42
Proportion incorrect	0.72	0.73	0.66	0.58	0.21	0.58
Correct/incorrect	0.39	0.37	0.51	0.73	3.84	0.73

The table also shows the proportions as well as the ratio of correctly and incorrectly identified referents.

**Table 9:** **Homogeneous subsets for counts of *correctly identified referents***.

**Algorithm**				**Correct**	**Total**
Human	A			123	155
Alg2		B		63	149
Alg1		B	C	49	146
DBP			C	44	156
IA			C	41	151

Algorithms that do not share a letter are significantly different with p < 0.05.

The results show a difference between algorithms Alg2 and Alg1, suggesting that the content-based termination heuristic might increase the chances of successful identification. Note, however, that the difference was not statistically significant and more investigation is required to investigate this issue.

Table 10 shows mean *naturalness* and *quality* for each algorithm. While the differences in *naturalness* are small, the algorithms seem to differ substantially in terms of *quality*. Because each description was viewed 3 times, the data were aggregated so that the *naturalness* and *quality* ratings for each description were created by taking the mean of the 3 ratings. We performed two one-way analyses of variance with ALGORITHM as the independent variable. The main effect of ALGORITHM was significant on *naturalness* [*F*_(4, 495)_ = 4.576, *p* < 0.005] and on *quality* [*F*_(4, 495)_ = 40.23, *p* < 0.001]. Tables 11, 12 show homogeneous subsets for *quality* and *naturalness* calculated by *post-hoc* Tukey test. Algorithms that do not share a letter are statistically different with *p* < 0.05. As we can see, the human-produced descriptions were rated highest. The analysis suggests that descriptions produced by the new algorithms have higher “*quality*” than the ones produced by the IA and DBpedia.

**Table 10:** **Mean ratings and standard deviations for *quality* and *naturalness* for each algorithm in the final evaluation**.

	**DBP**	**IA**	**Alg1**	**Alg2**	**Human**
Mean quality	43.570	57.857	67.600	66.786	77.552
Quality SD	27.385	20.630	16.670	18.051	15.570
Mean naturalness	61.953	61.927	62.110	61.495	70.311
Naturalness SD	18.587	17.294	17.758	18.655	15.556

Quality refers to the statement: “Suppose you did not know this person, how good would you find the description?” and naturalness refers to the statement: “How natural does the description read to you?”

**Table 11:** **Homogeneous subsets for *quality* calculated using *post-hoc* Tukey test**.

**Algorithm**					**Mean quality**	***SD***
Human	A				77.6	15.6
Alg2		B			66.8	18.1
Alg1		B			67.6	16.7
IA			C		57.9	20.6
DBP				D	43.6	27.4

Algorithms that do not share a letter are significantly different at p < 0.05.

**Table 12:** **Homogeneous subsets for *naturalness* calculated using a *post-hoc* Tukey test**.

**Algorithm**			**Mean naturalness**	***SD***
Human	A		70.3	15.6
Alg2		B	61.5	18.7
Alg1		B	62.1	17.8
IA		B	61.9	17.3
DBP		B	62.0	18.6

Algorithms that do not share a letter are significantly different at *p* < 0.05

## 7. General discussion

Our computational model addresses what we believe to be an interesting variant of the much-studied problem of reference production. The model does a better job addressing its task—producing descriptions of famous people to an unknown audience—than the Incremental Algorithm, both in terms of the numbers of correctly identified referents and in terms of the perceived quality of the descriptions generated. The structure of the model differs sharply from earlier ones. This is not only true in comparison to the algorithms proposed in practical Computational Linguistics (of which Siddharthan et al., 2011, developed in a context of automatic text summarization, is a good example), but also in comparison to algorithms developed in the tradition of REG. To ensure that our contributions are understood properly, it is worth re-stating some features of our approach.

Although some previous models of referring were constructed for situations in which hearers know less than speakers (Garoufi and Koller, 2014b; Paraboni and van Deemter, 2014b), these models assume that *the speaker knows what the hearer knows*. In many situations, however, speakers do not possess this information, for example when a journalist writes a newspaper article or a scientist a journal paper. Our model targets situations of this kind, where the key to success lies in the model's ability to make an educated guess concerning the knowledge of the reader. Additionally, we argue that these situations require that a guess is made about what information is likely to be distinctive for the hearer, and how much information the reader is likely to require.

Our model differs from earlier REG models because all three heuristics composing our model make use of pre-existing open-source data, rather than information that is hand crafted by researchers interested in reference. We believe that this lends additional interest to our model, because hand-crafting might accidentally benefit some algorithms over others. The use of open-source data is now well established in Computational Linguistics, but it has not been applied to the generation of referring expressions before.

Note, furthermore, that some key features of existing REG algorithms do not feature in our model. For example, since termination cannot be based on the criterion most often employed in REG (namely, that all distractors have been removed), we have had to find a different approach to this problem (Section 4.3). Similar observations can be made about discriminatory power (DP), a concept that had to be combined with information retrieval techniques to make it applicable to a situation in which the set of distractors is not know, and modified in light of the frequencies found in our data.

Some difficult questions are worth raising briefly. First, does our model have psychological reality, or is it merely a *product model* in the sense of Section 2? On the one hand, it is clearly a product model, since our final evaluation (Section 5) looked only at the output of the model, disregarding the actual production process. On the other hand, our tests of the individual heuristics do suggest that human speakers would be able to carry out these tests. Consider the Knowledge Heuristic, for example. Speakers evidently do not use Google to perform the kind of tests of which this heuristic makes use. Yet it may not be entirely implausible that speakers encounter, over the course of their lives, a large amount of text that is in some ways similar to (a suitable part of) the world-wide web, consequently considering the web as a model of human knowledge might not be ridiculous. The Knowledge Heuristic is no perfect model of human speaking, but it may be our best tool for capturing one aspect of it. Similar things might be said about Unexpectedness and Termination.

Should the Knowledge Heuristic be seen as a model of Common Ground? The experiment in Section 4.1 did not look at deeper levels of epistemic embedding (as in the classic notion of Common Ground of Section 1): at most, this experiment established that the heuristic predicts (approximately) what hearers know. On the other hand, if it is true, as is generally assumed, that reference rests on Common Ground, then our final evaluation—which suggests that the descriptions generated are effective and at least somewhat natural—suggests that, despite its relative simplicity, the Knowledge Heuristic makes reasonable predictions concerning (communal) Common Ground itself.

An example may make this clearer. Suppose it is debatable who invented the printing press: most Americans believe that this was done by Mr. *X*, but most Chinese believe it was done by Mr. *Y*. An American speaker who addresses a Chinese audience might choose, politely, to refer to Mr. *Y* (i.e., the Chinese scientist) as “the inventor of the printing press.” However, if her Chinese audience knows the speaker to be American, then they might misunderstand this description as referring to Mr. *X* (the American scientist), because they know that this is who the speaker believes the inventor of the printing press to be. Perhaps the success of our model can be seen as confirmation of Clark's idea that *communal* Common Ground tells us something about “real” (i.e., epistemically complex) Common Ground, and not just about a speaker's assessment of a hearer's knowledge.

Even though we have focussed on the production of referring expressions, it appears to us that elements of our proposal can be put to other uses. Consider Common Ground, for instance. To the extent that our Knowledge Heuristic is able to predict what facts a member of a particular community is likely to know, it is potentially relevant for many areas, such as journalism, advertising, and creative writing, because in all these areas it is important to assess what an unknown hearer is likely to know.

In a computational setting, the Knowledge Heuristic is applicable to a key problem that arises in many Information Presentation systems, namely to decide what information should be provided to the user. In Natural Language Generation, this is known as the problem of general Content Selection (Reiter and Dale, 2000). This time the Knowledge Heuristic could be “used in reverse,” selecting information that is *least* (rather than most) likely to be known, hence most worthy of being added to the reader's store of information (cf., Section 2.3, where Information Sharing is discussed). Similar observations can be made about the other two factors explored in our model. For example, many Content Selection approaches in Natural Language Generation aim to select interesting information; our Unexpectedness Heuristics could help.

Having said this, we acknowledge that we have merely put the first step on the road to understanding how the different factors may be estimated. For example, one could test the performance of our heuristics in specialist domains, for instance involving an audience of experts in some area of public life (say, football, or ballet), if a corpus of texts representing the knowledge of this audience can be found. Likewise, it would be interesting to investigate how the model fares at describing companies or geographic locations (rather than famous people).

A difficult question is how our approach might generalize to more complex types of information. At least in its implementation detail, the approach is difficult to extend to logically complex information: it is one thing to search a set of documents for the fact that Ernest Hemingway was American, for instance, (an atomic fact) than to search for the fact that he wrote *three novels*, or that he wrote *more novels than short stories*. This information may well be part of the Common Ground of a given community, but our computational model is not yet able to find it.

On a final, theoretical note, our Knowledge Heuristic can be seen as a first step toward a computational model of Herb Clark's Communal Common Ground. Our approach suggests, moreover, that it might be useful to extend the notion of Common Ground beyond its original conception, taking into account not only what speakers and hearers know, but also what they are interested in. After all, in communication, the interlocutors' interests are as important as their knowledge.

## Funding

The research reported in this article is based on the Ph.D. project of Dr. RK, which was funded by the Scottish Informatics and Computer Science Alliance (SICSA). KvD acknowledges support from the EPSRC under the RefNet grant (EP/J019615/1).

### Conflict of interest statement

The authors declare that the research was conducted in the absence of any commercial or financial relationships that could be construed as a potential conflict of interest.

## Footnotes

^1^Additionally, as will be explained, this elicitation experiment will play a minor technical role in our modeling of the Termination factor.

^2^For example, the Incremental Algorithm of Dale and Reiter (1995) was inspired by Pechmann's findings regarding incrementality (Pechmann, 1989) and by Rosch's work on basic-level values (Rosch, 1978).

^3^Isaac Newton.

^4^http://www.bbc.co.uk/history/historic_figures/

^5^Investigation of the large number of incomplete answers and potential issues of data quality are addressed in Section 4.1.8.

^6^http://www.bbc.co.uk/history/historic_figures/

^7^www.biographyonline.net/people/famous-100.html and www.whoismorefamous.com/?fulllist=1 last retrieved 21st August 2015.
